# Biomedical ontology alignment: an approach based on representation learning

**DOI:** 10.1186/s13326-018-0187-8

**Published:** 2018-08-15

**Authors:** Prodromos Kolyvakis, Alexandros Kalousis, Barry Smith, Dimitris Kiritsis

**Affiliations:** 1 École Polytechnique Fédérale de Lausanne (EPFL), Route Cantonale, Lausanne, 1015 Switzerland; 2 Business Informatics Department, University of Applied Sciences, HES-SO, Western Switzerland Carouge, Switzerland; 3 Department of Philosophy and Department of Biomedical Informatics, 104 Park Hall, University at Buffalo, Buffalo, 14260 NY USA

**Keywords:** Ontology matching, Semantic similarity, Sentence embeddings, Word embeddings, Denoising autoencoder, Outlier detection

## Abstract

**Background:**

While representation learning techniques have shown great promise in application to a number of different NLP tasks, they have had little impact on the problem of ontology matching. Unlike past work that has focused on feature engineering, we present a novel representation learning approach that is tailored to the ontology matching task. Our approach is based on embedding ontological terms in a high-dimensional Euclidean space. This embedding is derived on the basis of a novel phrase retrofitting strategy through which semantic similarity information becomes inscribed onto fields of pre-trained word vectors. The resulting framework also incorporates a novel outlier detection mechanism based on a denoising autoencoder that is shown to improve performance.

**Results:**

An ontology matching system derived using the proposed framework achieved an F-score of 94% on an alignment scenario involving the Adult Mouse Anatomical Dictionary and the Foundational Model of Anatomy ontology (FMA) as targets. This compares favorably with the best performing systems on the Ontology Alignment Evaluation Initiative anatomy challenge. We performed additional experiments on aligning FMA to NCI Thesaurus and to SNOMED CT based on a reference alignment extracted from the UMLS Metathesaurus. Our system obtained overall F-scores of 93.2% and 89.2% for these experiments, thus achieving state-of-the-art results.

**Conclusions:**

Our proposed representation learning approach leverages terminological embeddings to capture semantic similarity. Our results provide evidence that the approach produces embeddings that are especially well tailored to the ontology matching task, demonstrating a novel pathway for the problem.

## Background

Ontologies seek to alleviate the Tower of Babel effect by providing standardized specifications of the intended meanings of the terms used in given domains. Formally, an ontology is “a representational artifact, comprising a taxonomy as proper part, whose representations are intended to designate some combinations of universals, defined classes and certain relations between them” [1]. Ideally, in order to achieve a unique specification for each term, ontologies would be built in such a way as to be non-overlapping in their content. In many cases, however, domains have been represented by multiple ontologies and there thus arises the task of *ontology matching*, which consists in identifying correspondences among entities (types, classes, relations) across ontologies with overlapping content.

Different ontological representations draw on the different sets of natural language terms used by different groups of human experts [2]. In this way, different and sometimes incommensurable terminologies are used to describe the same entities in reality. This issue, known as the *human idiosyncrasy* problem [1], constitutes the main challenge to discovering equivalence relations between terms in different ontologies.

Ontological terms are typically common nouns or noun phrases. According to whether they do or do not include prepositional clauses [3], the latter may be either composite (for example *Neck of femur*) or simple (for example *First tarsometatarsal joint* or just *Joint*). Such grammatical complexity of ontology terms needs to be taken into account in identifying semantic similarity. But account must be taken also of the ontology’s axioms and definitions, and also of the position of the terms in the ontology graph formed when we view these terms as linked together through the *is*_*a* (subtype), *part*_*of* and other relations used by the ontology.

The primary challenge to identification of semantic similarity lies in the difficulty we face in distinguishing true cases of similarity from cases where terms are merely “descriptively associated”^1^. As a concrete example, the word “harness” is descriptively associated with the word “horse” because a harness is often used on horses [4]. Yet the two expressions are not semantically similar. The sorts of large ontologies that are the typical targets of semantic similarity identification contain a huge number of such descriptively associated term pairs. This difficulty in distinguishing similarity from descriptive association is a well-studied problem in both cognitive science [5] and NLP [6].

Traditionally, feature engineering has been the predominant way to approach the ontology matching problem [7]. In machine learning, a feature is an individual measurable property of a phenomenon in the domain being observed [8]. Here we are interested in features of terms, for instance the number of incoming edges when a term is represented as the vertex of an ontology graph; or a terms’s tf-idf value – which is a statistical measure of the frequency of a term’s use in a corpus [9]. Feature engineering consists in crafting features of the data that can be used by machine learning algorithms in order to achieve specific tasks. Unfortunately determining which hand-crafted features will be valuable for a given task can be highly time consuming. To make matters worse, as Cheatham and Hitzler have recently shown, the performance of ontology matching based on such engineered features varies greatly with the domain described by the ontologies [10].

As a complement to feature engineering, attempts have been made to develop machine-learning strategies for ontology matching based on binary classification [11]. This means a classifier is trained on a set of alignments between ontologies in which correct and incorrect mappings are identified with the goal of using the trained classifier to predict whether an assertion of semantic equivalence between two terms is or is not true. In general, the number of true alignments between two ontologies is several orders of magnitude smaller than the number of all possible mappings, and this introduces a serious class imbalance problem [12]. This abundance of negative examples hinders the learning process, as most data mining algorithms assume balanced data sets and so the classifier runs the risk of degenerating into a series of predictions to the effect that every alignment comes to be marked as a misalignment.

Both standard approaches thus fail: feature engineering because of the failure of generalization of the engineered features, and supervised learning because of the class imbalance problem. Our proposal is to address these limitations through the exploitation of unsupervised learning approaches for ontology matching drawing on the recent rise of distributed neural representations (DNRs), in which for example words and sentences are embedded in a high-dimensional Euclidean space [13–17] in order to provide a means of capturing lexical and sentence meaning in an unsupervised manner. The way this works is that the machine learns a mapping from words to high-dimensional vectors which take account of the contexts in which words appear in a plurality of corpora. Vectors of words that appear in the same sorts of context will then be closer together when measured by a similarity function. That the approach can work without supervision stems from the fact that meaning capture is merely a positive externality of context identification, a task that is unrelated to the meaning discovery task.

Traditionally, corpus driven approaches were based on the *distributional hypothesis*, i.e. the assumption that semantically similar or related words appear in similar contexts [18]. This meant that they tended to learn embeddings that capture both similarity (*horse, stallion*) and relatedness (*horse, harness*) reasonably well, but do very well on neither [6, 19]. In an effort to correct for these biases a number of pre-trained word vector refining techniques were introduced [6, 20, 21]. These techniques are however restricted to retrofitting single words and do not easily generalize to the sorts of nominal phrases that appear in ontologies. Wieting et al. [22, 23] make one step towards addressing the task of tailoring phrase vectors to the achievement of high performance on the semantic similarity task by focusing on the task of paraphrase detection. A paraphrase is a restatement of a given phrase that use different words while preserving meaning. Leveraging what are called universal compositional phrase vectors [24] for the purposes of paraphrase detection provides training data for the task of semantic similarity detection which extends the approach from single words to phrases. Unfortunately, the result still fails as regards the problem of distinguishing semantic similarity and descriptive association on rare phrases [22] – constantly appearing on ontologies – which thus again harms performance in ontology matching tasks.

In this work, we tackle the aforementioned challenges and introduce a new framework for representation learning based ontology matching. Our ontology matching algorithm is structured as follows: To represent the nouns and noun-phrases in an ontology, we exploit the context information that accompanies the corresponding expressions when they are used both inside and outside the ontology. More specifically, we create vectors for ontology terms on the basis of information extracted not only from natural language corpora but also from terminological and lexical resources and we join this with information captured both explicitly and implicitly from the ontologies themselves. Thus we capture contexts in which words are used in definitions and in statements of synonym relations. We also draw inferences from the ontological resources themselves, for example to derive statements of descriptive association – the absence of a synonymous statement between two terms with closely similar vectors is taken to imply that as a statement of descriptive association obtains between them. We then cast the problem of ontology matching as an instance of the Stable Marriage problem [25] discovering in that way terminological mappings in which there is no pair of terms that would rather be matched to each other than their current matched terms. In order to compute the ordering of preferences for each term, that the Stable Marriage problem requires, we use the terminological representations’ pairwise distances. We compute the aforementioned distances using the cosine distance over the phrases representations learned by the phrase retrofitting component. Finally, an outlier detection component sifts through the list of the produced alignments so as to reduce the number of misalignments.

Our main contributions in this paper are: (i) We demonstrate that word embeddings can be successfully harnessed for ontology matching; a task that requires phrase representations tailored to semantic similarity. This is achieved by showing that knowledge extracted from semantic lexicons and ontologies can be used to inscribe semantic meaning on word vectors. (ii) We additionally show that better results can be achieved on the discrimination task between semantic similarity and descriptive association, by casting the problem as an outlier detection. To do so, we present a denoising autoencoder architecture, which implicitly tries to discover a hidden representation tailored to the semantic similarity task. To the best of our knowledge, the overall architecture used for the outlier detection as well as its training procedure is applied for the first time to the problem of discriminating among semantically similar and descriptively associated terms. (iii) We use the biomedical domain as our application, due to its importance, its ontological maturity, and to the fact that it constitutes the domain with the larger ontology alignment datasets owing to its high variability in expressing terms. We compare our method to state-of-the-art ontology matching systems and show significant performance gains. Our results demonstrate the advantages that representation learning bring to the problem of ontology matching, shedding light on a new direction for a problem studied for years in the setting of feature engineering.

## Problem formulation

Before we proceed with the formal definition of an ontological entity alignment, we will introduce the needed formalism. Let *O*, *O*^′^ denote two set of terms used in two distinct ontologies and let *R* be a set of binary relations’ symbols. For instance, =,≠,*is*_*a* can be some of the *R* set’s citizens. We introduce a set *T*={(*e,r,e*^′^)|*e*∈*O,e*^′^∈*O*^′^,*r*∈*R*} to denote a set of possible binary relations between *O* and *O*^′^ [26]. Moreover, let $f \colon T \to [\!0,1] \subset \mathcal {R}$ be a function, called “confidence function”, that maps an element of *T* to a real number *v*, such that 0≤*v*≤1. The real number *v* corresponds to the degree of confidence that exists a relation *r* between *e* and *e*^′^ [27].

We call a set *T* of possible relations to be “valid despite integration inconsistency”, iff *T* is satisfiable. As an counterexample, the set {(*e*,=,*e*^′^),(*e*,≠,*e*^′^)} corresponds to a non-valid despite integration inconsistency set of relations. It should be noted that we slightly differentiated from the notation used in Description Logics [28], where a relation (Role) between two entities is denoted as: *r*(*e,e*^′^). Moreover, it is important to highlight the role of the phrase “despite integration inconsistency” in our definition. The ontology resulting from the integration of two ontologies *O* and *O*^′^ via a set of alignments *T* may lead to semantic inconsistencies [29, 30]. As the focus of ontology alignment lays on the discovery of alignments between two ontologies, we treat the procedure of inconsistency check as a process that starts only after the end of the ontology matching process^2^.

**Fig. 1:**
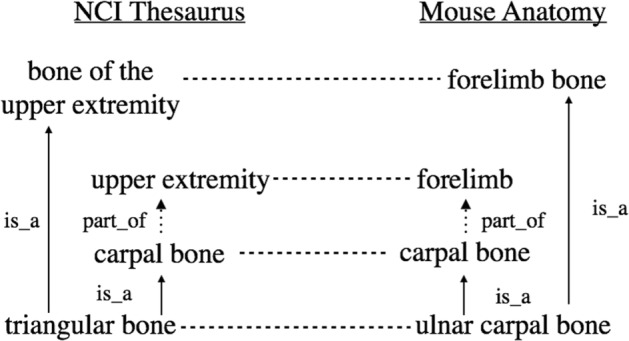
Example of alignments between the NCI Thesaurus and the Mouse Ontology (adapted from [56]). The dashed horizontal lines correspond to equivalence matchings between the NCI Thesaurus and the Mouse Anatomy ontology

Based on the aforementioned notations and definitions, we will proceed with the formal definition of what an ontological entity alignment is. Let, *T* be a valid despite integration inconsistency set of relations and *f* be a confidence function defined over *T*. Let (*e,r,e*^′^)∈*T*, we define an ontological entity correspondence between two entities *e*∈*O* and *e*^′^∈*O*^′^ as the four-element tuple:

1 $$  cor_{r}(e,e^{\prime}) = (e, r, e^{\prime}, f(e, r, e^{\prime}))  $$

where *r* is a matching relation between *e* and *e*^′^ (e.g., equivalence, subsumption) and *f*(*e, r, e*^′^)∈ [ 0,1] is the degree of confidence of the matching relation between *e* and *e*^′^. According to the examples presented in Fig. 1, (triangular bone,=,ulnar carpal bone,1.00) and (triangular bone,*is*_*a*,forelimb bone,1.00) present one equivalence as well as a subsumption entity correspondence, accordingly. In this work, we focus on discovering one-to-one equivalence correspondences between two ontologies. In absence of further relations, the produced set of relations by our algorithm will always correspond to a valid despite integration inconsistency set.

## System architecture overview

Our ontology matching system is composed of two neural network components that learn which term alignments correspond to semantic similarity. The first component discovers a large amount of true alignments between two ontologies but is prone to errors. The second component corrects these errors. We present below an overview of the two components.

The first component, which we call *phrase retrofitting* component, retrofits word vectors so that when they are used to represent sentences, the produced sentence embeddings will be tailored to semantic similarity. To inscribe semantic similarity onto the sentence embeddings, we construct an optimization criterion which rewards matchings of semantically similar sentence vectors and penalizes matchings of descriptively associated ones. Thus the optimization problem adapts word embeddings so that they are more appropriate to the ontology matching task. Nonetheless, one of the prime motivations of our work comes from the observation that although supervision is used to tailor phrase embeddings to the task of semantic similarity, the problem of discriminating semantically similar vs descriptively associated terms is not targeted directly. This lack will lead to the presence of a significant number of misalignments, hindering the performance of the algorithm.

For that reason, we further study the discrimination problem in the setting of unsupervised outlier detection. We use the set of sentence representations produced by the phrase retrofitting component to train an denoising autoencoder [31]. The denoising autoencoder (DAE) aims at deriving a hidden representation that captures intrinsic characteristics of the distribution of semantically similar terms. We force the DAE to leverage new sentence representations by learning to reconstruct not only the original sentence but also its paraphrases, thus boosting the semantic similarity information that the new representation brings. Since we are using paraphrases to do so we bring in additional training data, doing essentially data augmentation for the semantically similar part of the problem. The DAE corresponds to our second component which succeeds in discovering misalignments by capturing intrinsic characteristics of semantically similar terms.

## Methods

We present a representation learning based ontology matching algorithm that approaches the problem as follows: We use the ontologies to generate negative training examples that correspond to descriptively associated examples, and additional knowledge sources to extract paraphrases that will correspond to positive examples of semantic similarity. We use these training data to refine pre-trained word vectors so that they are better suited for the semantic similarity task. This task is accomplished by the phrase retrofitting component. We represent each ontological term as the bag of words of its textual description^3^ which we complement with the refined word embeddings. We construct sentence representations of the terms’ textual description by averaging the phrase’s aforementioned word vectors. We match the entities of two different ontologies using the Stable Marriage algorithm over the terminological embeddings’ pairwise distances. We compute the aforementioned distances using the cosine distance. Finally, we iteratively pass through all the produced alignments and we discard those that violate a threshold which corresponds to an outlier condition. We compute the outlier score using the cosine distance over the features created by an outlier detection mechanism.

### Preliminaries

We introduce some additional notation that we will use throughout the paper. Let $sen_{i} = \left \{w_{1}^{i}, w_{2}^{i}, \dots, w_{m}^{i} \right \}$ be the phrasal description^3^ of a term *i* represented as a bag of *m* word vectors. We compute the sentence representation of the entity *i*, which we denote *s*_*i*_, by computing the mean of the set *sen*_*i*_, as per [24]. Let $s_{i}, s_{j} \in \mathbb {R}^{d}$ be two *d*-dimensional vectors that correspond to two sentence vectors, we compute their cosine distance as follows: *dis*(*s*_*i*_,*s*_*j*_)=1−*cos*(*s*_*i*_,*s*_*j*_). In the following, *d* will denote the dimension of the pre-trained and retrofitted word vectors. For $x \in \mathbb {R}$, we denote the *rectifier* activation function as: *τ*(*x*)= max(*x*,0), and the *sigmoid* function as: $\sigma (x) = \frac {1}{1+e^{-x}}$.

### Building sentence representations

In this section, we describe the neural network architecture that will produce sentence embeddings tailored to semantic similarity. Quite recently several works addressed the challenge of directly optimizing word vectors to produce sentence vectors by averaging the bag of the word vectors [22, 32, 33]. The interplay between semantical and physical intuition is that word vectors can be thought as corresponding to the positions of equally weighted masses, where the center of their masses provides information of the mean location of their semantic distribution. Intuitively, the word vectors’ “center of the mass” provide a means for measuring where the semantic content primarily “concentrates”. Despite the fact that vector addition is insensitive to word order [24], it has been proven that this syntactic agnostic operation provides results that compete favorably with more sophisticated syntax-aware composing operations [33]. We base our phrase retrofitting architecture on an extension of the Siamese CBOW model [32]. The fact that Siamese CBOW provides a native mechanism for discriminating between sentence pairs from different categories explains our choice to build upon this architecture.

Siamese CBOW is a log linear model aiming at predicting a sentence from its adjacent sentences; addressing the research question whether directly optimizing word vectors for the task of being averaged leads to better suited word vectors for this task compared to word2vec [15]. Let *V*={*v*_1_,*v*_2_,…*v*_*N*_} be an indexed set of word vectors of size *N*. The Siamese CBOW model transforms a pre-trained vector set *V* into a new one, $V^{\prime } = \left \{v^{\prime }_{1}, v^{\prime }_{2}, \dots v^{\prime }_{N}\right \}$, based on two sets of positive, $S_{i}^{+}$, and negative, $S_{i}^{-}$, constraints for a given training sentence *s*_*i*_. The supervised training criterion in Siamese CBOW rewards co-appearing sentences while penalizing sentences that are unlikely to appear together. Sentence representations are computed by averaging the sentence’s constituent word vectors. The reward is given by the pairwise sentence cosine similarity over their learned vectors. Sentences which are likely to appear together should have a high cosine similarity over their learned representations. In the initial paper of Siamese CBOW [32], the set $S_{i}^{+} $ corresponded to sentences appearing next to a given *s*_*i*_, whereas $S_{i}^{-} $ corresponded to sentences that were not observed next to *s*_*i*_.

Since we want to be able to differentiate between semantically similar and descriptively associated sentences we let the sets $S_{i}^{+}$ and $S_{i}^{-}$ to be sentences that are semantically similar and descriptively associated to a given sentence *s*_*i*_. In the rest of the section we revise the main elements of the Siamese CBOW architecture and describe the modifications we performed in order to exploit it for learning sentence embeddings that reflect semantic similarity. To take advantage of the semantic similarity information already captured in the initial word vectors, an important characteristic as demonstrated in various word vectors retrofitting techniques [20–22], we use *knowledge distillation* [34] to penalize large changes in the learned word vectors with regard to the pre-trained ones.

Our paraphrase retrofitting model retrofits a pre-trained set of word vectors with the purpose of leveraging a new set *V*^′^, solving the following optimization problem:

2 $$ { \min \limits_{V^{\prime}} \kappa_{S} L_{S}(V^{\prime}) + \kappa_{LD} L_{KD}(V,V^{\prime}), }  $$

where *k*_*S*_ and *k*_*LD*_ are hyperparameters controlling the effect of *L*_*S*_(*V*^′^) and *L*_*KD*_(*V,V*^′^) losses, accordingly. The *L*_*S*_(*V*^′^) term is defined as $\frac {1}{N}\sum _{i=1}^{N}L_{S_{i}}$, where *N* denotes the number of the training examples. The $L_{S_{i}}$ term corresponds to categorical cross-entropy loss defined as:

3 $$ L_{S_{i}} = - \sum_{s_{j} \in \left\{ S_{i}^{+} \thinspace\cup\thinspace S_{i}^{-} \right\}} p(s_{i}, s_{j}) \cdot \log(p_{\theta}(s_{i}, s_{j})),  $$

where *p*(·) is the target probability the network should produce, and *p*_*θ*_(·) is the prediction it estimates based on parameters *θ*, using Eq. 5. The target distribution simply is:

4 $$ p(s_{i}, s_{j}) = \left\{ \begin{array}{ll} \frac{1}{|S^{+}|}, & \text{if}\ s_{j} \in S_{i}^{+} \\ 0, & \text{if}\ s_{j} \in S_{i}^{-}. \end{array} \right.  $$

For instance, if there are two positive and two negative examples, the target distribution is (0.5, 0.5, 0, 0). For a pair of sentences (*s*_*i*_,*s*_*j*_), the probability *p*_*θ*_(*s*_*i*_,*s*_*j*_) is constructed to reflect how likely it is for the sentences to be semantically similar, based on the model parameter *θ*. The probability *p*_*θ*_(*s*_*i*_,*s*_*j*_) is computed on the training data set based on the softmax function as follows:

5 $$ p_{\theta}(s_{i}, s_{j}) = \frac{e^{{\left(\cos\left(\mathbf{s_{i}^{\theta}}, \mathbf{s_{j}^{\theta}}\right)\right)}^{1/T}}}{\sum_{s_{k} \in\left \{ S_{i}^{+} \thinspace\cup\thinspace S_{i}^{-} \right\}} e^{{\left({\cos\left(\mathbf{s_{i}^{\theta}}, \mathbf{s_{k}^{\theta}}\right)}\right)}^{1/T}}},  $$

where $s^{\theta }_{x}$ denotes the embedding of sentence *s*_*x*_, based on the model parameter *θ*. To encourage the network to better discriminate between semantically similar and descriptively associated terms, we extend the initial architecture by introducing the parameter *T*. The parameter *T*, named *temperature*, is based on the recent work of [34, 35]. Hinton et al. [34] suggest that setting *T*>1 increases the weight of smaller logit (the inputs of the softmax function) values, enabling the network to capture information hidden in small logit values.

To construct the set *S*^−^, we sample a set of descriptively associated terms from the ontologies to be matched. Given a sentence *s*_*i*_, we compute its cosine distance with every term from the two ontologies to be matched, based on the initial pre-trained word vectors. Thereafter, we choose the *n* terms demonstrating the smaller cosine distance to be the negative examples. To account for that fact that among these *n* terms there may be a possible alignment, we exclude the *n*_∗_ closest terms. Equivalently, given the increasingly sorted sequence of the cosine distances, we choose the terms in index positions starting from *n*_∗_ up to *n*+*n*_∗_. For computationally efficiency, we carry this process out only once before the training procedure starts.

Hinton et al. [34] found that using the class probabilities of an already trained network as “soft targets” for another one network constitutes an efficient way of communicating already discovered regularities to the latter network. We exploit, thus, knowledge distillation to emit the original semantic information captured in the pre-trained word vectors to the new ones leveraged by Siamese CBOW. Therefore, we add the Knowledge Distillation loss $L_{KD}(V,V^{\prime }) = \frac {1}{N}\sum _{i=1}^{N}L_{KD_{i}} $ to the initial Siamese CBOW’s loss. The $L_{KD_{i}}$ term:

6 $$ L_{KD_{i}} = - \sum_{s_{j} \in \{ S^{+} \thinspace\cup\thinspace S^{-} \}} p_{\theta_{I}}(s_{i}, s_{j}) \cdot \log(p_{\theta}(s_{i}, s_{j})),  $$

is defined as the categorical cross-entropy between the probabilities obtained with the initial parameters (i.e. *θ*_*I*_) and the ones with parameters *θ*.

**Fig. 2:**
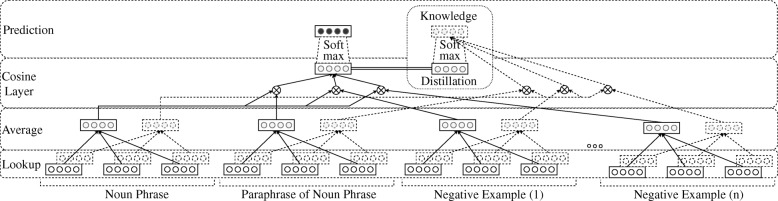
Phrase Retrofitting architecture based on a Siamese CBOW network [32] and Knowledge Distillation [34]. The input projection layer is omitted

Based on the observations of Hinton et al. [34], these “soft targets” act as an implicit reguralization, guiding the Siamese CBOW’s solution closer to the initial word vectors. We would like to highlight that we experimented with various regularizers, such as the ones presented in the works of [20, 21, 23, 36], however, we obtained worse results than the ones reported in our experiments. Figure 2 summarizes the overall architecture of our phrase retrofitting model. The dashed rectangles in the *Lookup Layer* correspond to the initial word vectors, which are used to encourage the outputs of the Siamese CBOW network to approximate the outputs produced with the pre-trained ones in every epoch. The word embeddings are averaged in the next layer to produce sentence representations. The cosine similarities between the sentence representations are calculated in the penultimate layer and are used to feed a softmax function so as to produce a final probability distribution. Specifically, we compute the cosine similarity between the sentence representation of the noun phrase and the sentence representations of every positive and negative example of semantic similarity. In the final layer, this probability distribution is used to compute two different categorical cross entropy losses. The left loss encourages the probability distribution values to approximate a target distribution, while the right one penalizes large changes in the learned word vectors with regard to the pre-trained ones. The double horizontal lines in the *Cosine Layer* highlight that these rectangles denote in fact the same probability distribution, computed in the penultimate layer.

### Outlier detection

The extension of the Siamese CBOW network retrofits pre-trained word vectors to become better suited for constructing sentence embeddings that reflect semantic similarity. Although we sample appropriate negative examples (i.e., descriptively associated terms) from the ontologies to be matched, we will never have all the negative examples needed. Moreover, allowing a larger number, *n*, of negative examples increases the computation needed making it inefficient. We depart from these problems by further casting the problem of discriminating between semantically similar and related terms as an outlier detection. To leverage an additional set of sentence representations more robust to semantic similarity, we use the hidden representation of a Denoising Autoencoder (DAE) [31].

The Siamese CBOW network learns to produce sentence embeddings of ontological terms that are better suited for the task of semantic similarity. We now use the learned sentence vectors to train a DAE. We extend the *standard* architecture of DAEs to reconstruct not only the sentence representation fed as input but also paraphrases of that sentence. Our idea is to improve the sentence representations produced by the Siamese CBOW and make them more robust to paraphrase detection. At the same time, this constitutes an efficient data augmentation technique; very important in problems with relatively small training data sets.

We train the autoencoder once the training of the Siamese CBOW network has been completed. Even if layer-wise training techniques [37] are outweighed nowadays by end-to-end training, we decide to adopt this strategy for two reasons. Firstly, we aim to capture with the DAE intrinsic characteristics of the distribution of the semantically similar terms. DAEs have been proven to really capture characteristics of the data distribution, namely the derivative of the log-density with respect to the input [38]. However, training the DAE on a dataset that does not reflect the true distribution of semantically similar terms introduces surely a barrier to our attempt. Therefore, we leverage in advance sentence representations, through the Siamese CBOW network, more robust to semantic similarity; an action that allows the DAE to act on a dataset with significantly less noise and less bias. Secondly, combining the extended Siamese CBOW architecture together with the DAE and training them end-to-end significantly increases the number of the training parameters. This increase is a clear impediment to a problem lacking an oversupply of training data.

Let $x, y \in \mathbb {R}^{d}$ be two *d*-dimensional vectors, representing the sentence vectors of two paraphrases. Our target is not only to reconstruct the sentence representation from a corrupted version of it, but also to reconstruct a paraphrase of the sentence representation based on the partial destroyed one. The corruption process that we followed in our experiments is the following: for each input *x*, a fixed number of *vd* (0<*v*<1) components are chosen at random, and their value is forced to 0, while the others are left untouched. The corrupted input $\tilde {x}$ is then mapped, as with the basic autoencoder, to a hidden representation $h = \tau (W\tilde {x}+b)$ from which we reconstruct a *z*=*σ*(*W*^′^*h*+*b*^′^). The dimension *d*_*h*_ of the hidden representation $h \in \mathbb {R}^{d_{h}}$ is treated as a hyperparameter. Similar to the work in [31], the parameters are trained to minimize, over the training set, an average reconstruction error. However, we aim not only to reconstruct the initial sentence but also its paraphrases. For that reason, we use the following reconstruction loss: *L*(*x,z*)+*L*(*y,z*)=

7 $$ \begin{array}{ll} = & - \sum\limits_{k=1}^{d} [\! x_{k} \log z_{k} + (1-x_{k})\log (1-z_{k}) ] \\ & - \sum\limits_{k=1}^{d} [\! y_{k} \log z_{k} + (1-y_{k})\log (1-z_{k}) ]. \end{array}  $$

**Fig. 3:**
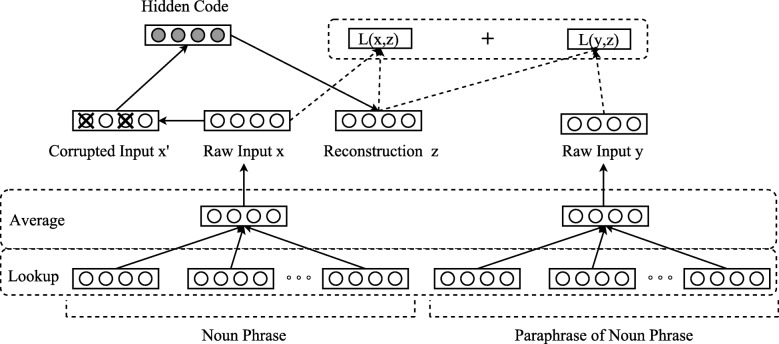
Autoencoder Architecture

The *x*_*k*_, *z*_*k*_, *y*_*k*_ correspond to the Cartesian coordinates of vectors *x*, *z* and *y*, respectively. The overall process is depicted in Fig. 3. In this figure, the Lookup and Average layers are similar to the ones depicted in Fig. 2. A sentence representation *x* is corrupted to $\tilde {x}$. The autoencoder maps it to *h* (i.e., the hidden code) and attempts to reconstruct both *x* and the paraphrase embedding *y*.

### Ontology matching

**Fig. 4:**
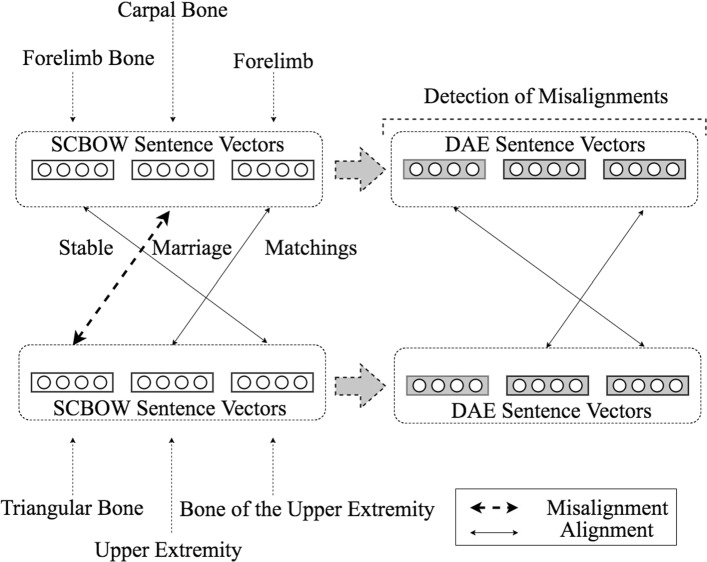
Overall proposed ontology matching architecture

The two components that we have presented were build in such a way so that they learn sentence representations which try to disentagle semantic similarity and descriptive association. We will now use these representations to solve the ontology matching problem (Fig. 4). To align the entities from two different ontologies, we use the extension of the Stable Marriage Assignment problem to unequal sets [25, 39]. This extension of the stable marriage algorithm computes 1−1 mappings based on a preference *m*×*n* matrix, where m and n is the number of entities in ontologies *O* and *O*^′^, respectively. In our setting, a matching is not stable if: (i) there is an element *e*_*i*_∈*O* which prefers some given element *e*_*j*_∈*O*^′^ over the element to which *e*_*i*_ is already matched, and (ii) *e*_*j*_ also prefers *e*_*i*_ over the element to which *e*_*j*_ is already matched. These properties of a stable matching impose that it does not exist any match (*e*_*i*_,*e*_*j*_) by which both *e*_*i*_ and *e*_*j*_ would be individually matched to more similar entities compared to the entities to which they are currently matched. This leads to a significant reduction in the number of misalignments due to descriptive association, provided that the learned representations do reflect the semantic similarity.

The steps of our ontology matching algorithm are the following: We represent each ontological term as the bag of words of its textual description, which we complement with the refined word vectors produced by the phrase retrofitting component. In the next step, we construct phrase embeddings of the terms’ textual description^3^ by averaging the phrase’s word vectors. We cast the problem of ontology matching as an instance of the Stable Marriage problem using the entities’ semantic distances. We compute these distances using the cosine distance over the sentences vectors. We iteratively pass through all the produced alignments and we discard those with a cosine distance greater than a certain threshold, *t*_1_. These actions summarize the work of the first component. Note that the violation of the triangle inequality by the cosine distance is not an impediment to the Stable Marriage algorithm [25].

In the next step, we create an additional set of phrase vectors by passing the previously constructed phrase vectors through the DAE architecture. Based on this new embedding’s set, we iteratively pass through all the alignments produced in the previous step and we discard those that report a threshold violation. Specifically, we discard those that exhibit a cosine distance, computed over the vectors produced by the DAE, greater than a threshold *t*_2_. This corresponds to the final step of the outlier detection process as well as of our ontology matching algorithm.

## Results and discussion

In this section, we present the experiments we performed on biomedical evaluation benchmarks coming from the Ontology Alignment Evaluation Initiative (OAEI), which organizes annual campaigns for evaluating ontology matching systems. We have chosen the biomedical domain for our evaluation benchmarks owing to its ontological maturity and to the fact that its language use variability is exceptionally high [40]. At the same time, the biomedical domain is characterized by rare words and its natural language content is increasing at an extremely high speed, making hard even for people to manage its rich content [41]. To make matters worse, as it is difficult to learn good word vectors for rare words from only a few examples [42], their generalization on their ontology matching task is questionable. This is a real challenge for domains, such as the biomedical, the industrial, etc, in which existence of words with rare senses is typical. The existence of rare words makes the presence of the phrase retroffiting component crucial to the performance of our ontology alignment framework.

### Biomedical ontologies

We give a brief overview of the four ontologies used in our ontology mapping experiments. Two of them (the Foundational Model of Anatomy and the Adult Mouse anatomical ontologies) are pure anatomical ontologies, while the other two (SNOMED CT and NCI Thesaurus) are broader biomedical ontologies of which anatomy consists a subdomain that they describe [3]. Although more recent versions of these resources are available, we refer to the versions that appear in the Ontology Alignment Evaluation Initiative throughout this work in order to facilitate comparisons across the ontology matching systems.

**Foundational Model of Anatomy (FMA):** is an evolving ontology that has been under development at the University of Washington since 1994 [43, 44]. Its objective is to conceptualize the phenotypic structure of the human body in a machine readable form.

**Adult Mouse Anatomical Dictionary (MA):** is a structured controlled vocabulary describing the anatomical structure of the adult mouse [45].

**NCI Thesaurus (NCI)** provides standard vocabularies for cancer [46] and its anatomy subdomain describes naturally occurring human biological structures, fluids and substances.

**SNOMED Clinical Terms (SNOMED):** is a systematically organized machine readable collection of medical terms providing codes, terms, synonyms and definitions used in clinical documentation and reporting [47].

### Semantic lexicons

We provide below some details regarding the procedure we followed in order to construct pairs of semantically similar phrases. Let $(word_{1}^{1}, word_{2}^{1}, \dots, word_{m}^{1})$, be a term represented as a sequence of *m* words. The strategy that we have followed in order to create the paraphrases is the following: We considered all the contiguous subsequences of this term. Namely, we considered all the possible contiguous subsequences of the form: $(word_{i}^{1}, word_{(i+1)}^{1}, \dots, word_{j}^{1})$, $\forall i, j \in \mathbb {N}: 0 \leq i \leq j \leq m$. Based on these contiguous subsequences, we queried the semantic lexicons for paraphrases. Below we give a brief summary of the semantic lexicons that we used in our experiments:

**ConceptNet 5:** a large semantic graph that describes general human knowledge and how it is expressed in natural language [48]. The scope of ConceptNet includes words and common phrases in any written human language.

**BabelNet:** a large, wide-coverage multilingual semantic network [49, 50]. BabelNet integrates both lexicographic and encyclopedic knowledge from WordNet and Wikipedia.

**WikiSynonyms:** a semantic lexicon which is built by exploiting the Wikipedia redirects to discover terms that are mostly synonymous [51].

Apart from the synonymy relations found in these semantic lexicons, we have exploited the fact that in some of the considered ontologies, a type may have one preferred name and some additional paraphrases [3], expressed through multiple rdfs:label relations.

### Training

We tuned the hyperparameters on a set of 1000 alignments which we generated by subsampling the SNOMED-NCI ontology matching task^4^. We chose the vocabulary of the 1000 alignments so that it is disjoint to the vocabulary that we used in the alignment experiments, described in the evaluation benchmarks, in order to be sure that there is no information leakage from training to testing. We tuned to maximize the *F*1 measure. We trained with the following hyperparameters: word vector has size (*d*) 200 and is shared across everywhere. We initialized the word vectors from word vectors pre-trained on a combination of PubMed and PMC texts with texts extracted from a recent English Wikipedia dump [52]. All the initial out-of-vocabulary word vectors are sampled from a normal distribution (*μ*=0,*σ*^2^=0.01). The resulted hyperparameters controlling the effect of retrofitting *k*_*S*_ and knowledge distillation *k*_*LD*_ were 10^6^ and 10^3^, accordingly. The resulted size of the DAE hidden representation (*d*_*h*_) is 32 and *v* is set to 0.4. We used *T*=2 according to a grid search, which also aligns with the authors’ recommendations [34]. For the initial sampling of descriptively associated terms, we used: *n*_∗_=2 and *n*=7. The best resulted values for the thresholds were the following: *t*_1_=*t*_2_=0.2. The phrase retrofitting model was trained over 15 epochs using the Adam optimizer [53] with a learning rate of 0.01 and gradient clipping at 1. The DAE was trained over 15 epochs using the Adadelta optimizer [54] with hyperparameters *ε*=1*e*−8 and *ρ*=0.95.

### Evaluation benchmarks

We provide some details regarding the respective size of each ontology matching task on Table 1.

**Table 1:** Respective sizes of the ontology matching tasks

Ontology Matching between:				#Matchings
Ontology I	#Types	Ontology II	#Types	
MA	2744	NCI	3304	1489
FMA	3696	NCI	6488	2504
FMA	10157	SNOMED	13412	7774

The reference alignment of the MA - NCI matching scenario is based on the work of Bodenreider et al. [55]. To represent each ontological term for this task, we used the unique rdfs:label that accompanies every type in the ontologies. The alignment scenarios between FMA - NCI and FMA - SNOMED are based on a small fragment of the aforementioned ontologies. The reference alignments of these alignment scenarios are based on the UMLS Metathesaurus [56], which currently consists the most comprehensive effort for integrating independently developed medical thesauri and ontologies. To represent each ontological term for these tasks, we exploited the textual information appearing on the rdf:about tag that accompanies every type in the ontologies. We did not use the rdf:about tag on the MA - NCI matching scenario, since their rdf:about tags provide a language agnostic unique identifier with no direct usable linguistic information. We would like to note that since the Stable Marriage algorithm provides one-to-one correspondences, we have only focused on discovering one-to-one matchings. In addition, a textual preprocessing that we performed led a small number of terms to degenerate into a single common phrase. This preprocessing includes case-folding, tokenization, removal of English stopwords and words coappearing in the vast majority of the terms (for example the word “structure” in SNOMED). Thereafter, we present on Table 1 the number of one-to-one types’ equivalences remained after the preprocessing step.

Last but not least, it is of significant importance to highlight that the reference alignments based on UMLS Metathesaurus will lead to an important number of logical inconsistencies [57, 58]. As our method does not apply reasoning, whether it produces or not incoherence-causing matchings is a completely random process. In our evaluation, we have chosen to also take into account *incoherence-causing* mappings. However, various concerns can be raised about the fairness of comparing against ontology matching systems that make use of automated alignment repair techniques [58, 59]. For instance, the state-of-the-art systems AML [60, 61], LogMap and LogMapBio [62], which are are briefly described in the next section, do employ automated alignment repair techniques. Our approach to use the original and incoherent mapping penalizes these systems that perform additional incoherence checks.

Nonetheless, our choice to include inconsistence mappings can be justified in the following way. First, it is a direct consequence of the fact that we approach the problem of Ontology Matching from the viewpoint of discovering semantically similar terms. A great number of these inconsistent mappings do correspond to semantically similar terms. Second, we believe that ontology matching can also be used as a curation process during the ontological (re)design phase so as to alleviate the possibility of inappropriate terms’ usage. The fact that two distinct truly semantically similar terms from two different ontologies lead to logical inconsistencies during the integration phase can raise an issue for modifying the source ontology [57]. Third, although ontologies constitute a careful attempt to ascribe the intended meaning of a vocabulary used in a target domain, they are error prone as every human artifact. Incoherence check lays on the assumption that both of the ontologies that are going to be matched are indeed error-free representational artifacts. We decide not to make this assumption.

Therefore, we have chosen to treat even the systems that employ automated alignment repair techniques error-prone. For that reason, we considered appropriate to report the performance of the aforementioned systems on the complete reference alignment in the next section. Nevertheless, we refer the reader to the [58] for details on the performance of these systems on incoherence free subsets of the reference alignment set. Under the assumption that the ontologies to be matched are error-free, it can be observed that the automated alignment repair mechanisms of these systems are extremely efficient; a fact that demonstrates the maturity and the robustness of these methods.

### Experimental results

Table 2 shows the performance of our algorithm compared to the six top performing systems on the evaluation benchmarks, according to the results published in OAEI Anatomy track (MA - NCI) and in the Large BioMed track (FMA-NCI, FMA-SNOMED)^5^. To check for the statistical significance of the results, we used the procedure described in [63]. The systems presented in Table 2 starting from the top of the table up to and including LogMapBio fall into the category of feature engineering^6^. CroMatcher [64], AML [60, 61] and XMap [65] perform ontology matching based on heuristic methods that rely on aggregation functions. FCA_Map [66, 67] uses Formal Concept Analysis [68] to derive terminological hierarchical structures that are represented as lattices. The matching is performed by aligning the constructed lattices taking into account the lexical and structural information that they incorporate. LogMap and LogMapBio [62] use logic-based reasoning over the extracted features and cast the ontology matching to a satisfiability problem. Some of the systems compute many-to-many alignments between ontologies. For a fair comparison of our system with them, we have also restricted these systems in discovering one-to-one alignments. We excluded the results of XMap for the Large BioMed track, because it uses synonyms extracted by the UMLS Metathesaurus. Systems that use the UMLS Metathesaurus as background knowledge will have a notable advantage since the Large BioMed track’s reference alignments are based on it.

**Table 2:** Performance of ontology matching systems across the different matching tasks.

System	MA - NCI			FMA-NCI			FMA-SNOMED		
	P	R	F1	P	R	F1	P	R	F1
AML	0.943	0.94	**0.941**	0.908	0.94	0.924	0.938	0.784	0.854
CroMatcher	0.942	0.912	0.927	-	-	-	-	-	-
XMap	0.924	0.877	0.9	-	-	-	-	-	-
FCA_Map	0.922	0.841	0.880	0.89	0.947	0.918	0.918	0.857	0.886
LogMap	0.906	0.850	0.878	0.894	0.930	0.912	0.933	0.721	0.814
LogMapBio	0.875	0.900	0.887	0.88	0.938	0.908	0.93	0.727	0.816
Wieting	0.804	0.879	0.839	0.840	0.857	0.849	0.867	0.851	0.859
Wieting+DAE(O)	0.952	0.871	0.909	0.909	0.851	0.879	0.929	0.832	0.878
SCBOW	0.847	0.917	0.881	0.899	0.895	0.897	0.843	0.866	0.855
SCBOW+DAE(O)	0.968	0.913	0.94	0.976	0.892	**0.932**	0.931	0.856	**0.892**

Note: Bold and underlined numbers indicate the best *F*1-score and the best precision on each matching task, respectively

We describe in the following the procedure that we followed in order to evaluate the performance of the various ontology matching systems. Since the incoherence-causing mappings were also taken into consideration, all the mappings marked as “?” in the reference alignment were considered as positive. To evaluate the discovery of one-to-one matchings, we clustered all the m-to-n matchings and we counted only once when any of the considered systems discovers any of the m-to-n matchings. Specifically, let *T*={(*e*,=,*e*^′^)|*e*∈*O,e*^′^∈*O*^′^} be a set of clustered m-to-n matchings. Once an ontology matching system discovers for the first time a (*e*,=,*e*^′^)∈*T*, we increase the number of the discovered alignments. However, whenever the same ontology matching system discovers an additional $(e_{*},=,e_{*}^{\prime }) \in T$, where $(e,=,e^{\prime }) \neq (e_{*},=,e_{*}^{\prime })$, we did not take this discovered matching into account. Finally, to evaluate the performance of AML, CroMatcher, XMap, FCA_MAP, LogMap, and LogMapBio, we used the alignments provided by OAEI 2016^5^ and applied the procedure described above to get their resulted performance.

To explore the performance details of our algorithm, we report in Table 2 its performance results with and without outlier detection. Moreover, we included experiments in which instead of training word embeddings based on our extension of the Siamese CBOW, we have used the optimization criterion presented in [23] to produce an alternative set of word vectors. As before, we present experiments on which we exclude our outlier detection mechanism and experiments on which we allow it^7^. We present these experiments under the listings: SCBOW, SCBOW+DAE(O), Wieting, Wieting+DAE(O), accordingly.

SCBOW+DAE(O) is the top performing algorithm in two of the three ontology mappings tasks (FMA-NCI, FMA-SNOMED); in these two its F1 score is significantly better than that of all the other algorithms. In MA-NCA its F1 score is similar to AML, the best system there, but the performance difference is statistically significant. At the same time, SCBOW+DAE(O) achieves the highest precision on two out of three ontology matching tasks. In terms of recall, SCBOW+DAE(O) demonstrates lower performance in the ontology matching tasks. However, we would like to note that we have not used any semantic lexicons specific to the biomedical domains compared to the other systems. For instance, AML uses three sources of biomedical background knowledge to extract synonyms. Specifically, it exploits the Uber Anatomy Ontology (Uberon), the Human Disease Ontology (DOID), and the Medical Subject Headings (MeSH). Hence, our reported recall can be explained due to the lower coverage of biomedical terminology in the semantic lexicons that we have used. Our motivation for relying only on domain-agnostic semantic lexicons^8^ stems from the fact that our intention is to create an ontology matching algorithm applicable to many domains. The success of these general semantic lexicons for such a rich in terminology domain, provides additional evidence that the proposed methodology may also generalize to other domains. However, further experimentation is needed to verify the adequacy and appropriateness of these semantic lexicons to other domains. It is among our future directions to test the applicability of our proposed algorithm to other domains.

Comparing the recall^9^ of SCBOW and SCBOW+ DAE(O), we see that the incorporation of the DAE produces sentence embeddings that are tailored to the semantic similarity task. The small precision of SCBOW, in all experiments, indicates a semantic similarity and descriptive association coalescence. Considering both the precision and the recall metric, we can observe that the outlier detection mechanism identifies misalignments while preserving most of the true alignments. This fact provides empirical support on the necessity of the outlier detection. To validate the importance of our phrase retrofitting component, we further analyze the behavior of aligning ontologies based on the word embedding produced by running the procedure described in [23] (listed as Wieting). As we can see SCBOW achieves statistically significant higher recall than Wieting in all our experiments and in two of the three cases statistically significant greater precision. This behavior indicates the superiority of SCBOW in injecting semantic similarity to word embeddings as well as to produce word vectors tailored to the ontology matching task. We further extended the Wieting experiment by applying our outlier detection mechanism trained on the word vectors produced by the procedure described in [23]. It can be seen that this extension leads to the same effects as the ones summarized in the SCBOW - SCBOW+DAE(O) comparison. These results give evidence that our DAE-based outlier detection component constitutes a mechanism applicable to various sentence embeddings’ producing architectures.

### Ablation study

**Fig. 5:**
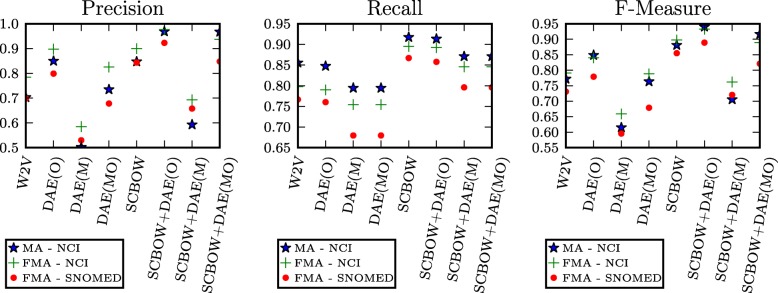
Feature ablation study of our proposed approach across all the experimental ontology matching tasks

In this section, an ablation study is carried out to investigate the necessity of each of the described components, as well as their effect on the ontology matching performance. Figure 5 shows a feature ablation study of our method; in Table 3 we give the descriptions of the experiments. We conducted experiments on which the phrase retrofitting component was not used, hence the ontology matching task was only performed based on the pre-trained word vectors. Moreover, we have experimented on performing the ontology matching task with the features generated by the DAE. Our prime motivation was to test whether the features produced by the DAE could be used to compute the cosine distances needed for estimating the preference matrix used by the Stable Marriage’s algorithm. Hence, we differentiate in this subsection and we allow the DAE features to be used for Matching and/or Outlier Detection.

**Table 3:** Ablation study experiment’s listings

Experiment’s code:	Phrase retrofitting	DAE features:	
		Matching	Outlier detection
W2V	-	-	-
DAE(O)	-	-	✓
DAE(M)	-	✓	-
DAE(MO)	-	✓	✓
SCBOW	✓	-	-
SCBOW+DAE(O)	✓	-	✓
SCBOW+DAE(M)	✓	✓	-
SCBOW+DAE(MO)	✓	✓	✓

To begin with, it can be observed that all the performance metrics’ figures undergo the same qualitative behavior. This result demonstrates that our algorithm exhibits a consistent behavior under the ablation study across all the experiments, which constitutes an important factor for inducing conclusions from experiments. The experiment W2V gives the results of executing the algorithm without the phrase retrofitting process, just by providing the pre-trained word vectors [52]. The performance of W2V in terms of Precision/Recall is systematically lower compared to all cases in which the initial word2vec vectors are retrofitted. These results support the importance of the phrase retrofitting process (experiments of which are presented under the listing SCBOW in Fig. 5), which succeeds in tailoring the word embeddings to the ontology matching task. The pre-trained word vectors, even though they were trained on PubMed and PMC texts, retain small precision and recall. This fact indicates a semantic similarity and descriptive association coalescence and sheds light on the importance of the retrofitting procedure.

Training the DAE on the pre-trained word vectors - DAE(O) - adds a significant performance gain on precision, which witnesses the effectiveness of the architecture for outlier detection. However, DAE(O)’s precision is almost the same as the one presented in the SCBOW experiment. Only when the phrase retrofitting component is combined with the DAE for outlier detection - SCBOW+DAE(O) - we manage to surpass the aforementioned precision value and achieve our best *F*1-score. Finally, our experiments on aligning ontologies by only using the DAE features demonstrate that these features are inadequate for this task. One prime explanation of this behavior is that DAE features are only exposed to synonymy information. At the same time, the dimensionality reduction of DAE features may lead them to lose a lot of valuable information captured in them for discriminating between semantically similar and descriptively associated terms. Note also that the preference matrix required by the Stable Marriage solution requires each term of an ontology *O* to be compared across all the possible terms of another ontology *O*^′^. Thereafter, the vectors based on which the preference matrix will be computed need to capture the needed information adequate for discriminating between semantically similar and descriptive associated terms.

### Error analysis

Recent studies provide evidence that different sentence representations objectives yield different intended representation preferable for different intended applications [33]. Moreover, our results reported in Table 2 on aligning ontologies with word vectors trained based on the method presented in [23] provide further evidence in the same direction. In Table 4, we demonstrate a sample of misalignments produced by aligning ontologies using the Stable Marriage’s solution based on a preference matrix computed either on SCBOW or Word2Vec vectors. It can be seen that the SCBOW misalignments demonstrate even a better spatial consistency compared to the Wor2Vec misalignments. This result combined with high *F*1-score reported in the SCBOW results in Table 5 show that ontological knowledge can be an important ally in the task of harnessing terminological embeddings tailored to semantic similarity. Moreover, this error analysis provides additional support for the significance of retrofitting general-purpose word embeddings before being applied in a domain-specific setting. It can be observed that general-purpose word vectors capture both similarity and relatedness reasonably well, but neither perfectly as it has been already observed in various works [6, 19].

**Table 4:** Sample misalignments produced by aligning ontologies using either SCBOW or Word2Vec vectors

Terminology to be matched	Matching based on SCBOW	Matching based on Word2Vec
MA-NCI		
gastrointestinal tract	digestive system	respiratory tract
tarsal joint	carpal tarsal bone	metacarpo phalangeal joint
thyroid gland epithelial tissue	thyroid gland medulla	prostate gland epithelium
FMA-NCI		
cardiac muscle tissue	heart muscle	muscle tissue
set of carpal bones	carpus bone	sacral bone
white matter of telencephalon	brain white matter	white matter
FMA-SNOMED		
zone of ligament of ankle joint	accessory ligament of ankle joint	entire ligament of elbow joint
muscle of anterior compartment of leg	compartment of lower leg	entire interosseus muscle of hand
dartos muscle	dartos layer of scrotum	tendon of psoas muscle

### Runtime analysis

In this section, we report the runtimes of our ontology matching algorithm for the different matching scenarios. Since our method – SCBOW+DAE(O) – consists of three major steps, we present in Table 5 the time devoted to each of them as well as their sum. In brief, the steps of our algorithm are the following: the training of the phrase retrofitting component (Step 1), the solution to the stable marriage assignment problem (Step 2), and finally the training of the DAE-based outlier detection mechanism (Step 3). All the reported experiments were performed on a desktop computer with an Intel ^®;^ Core ^™^ i7-6800K (3.60GHz) processor with 32GB RAM and two NVIDIA ^®;^ GeForce ^®;^ GTX ^™^ 1080 (8GB) graphic cards. The implementation was done in Python using Theano [69, 70].

**Table 5:** Runtimes of the steps in the proposed algorithm

Matching task	Running time (seconds)			
	Step 1	Step 2	Step 3	Total
MA - NCI	337	34	36	407
FMA - NCI	490	82	40	612
FMA - SNOMED	609	490	41	1140

As it can be seen on Table 5, the majority of the time is allotted to the training of the phrase retrofitting framework. In addition, it can be observed that the training overhead of the outlier detection mechanism is significantly smaller compared to the other steps. However, one important tendency can be observed in the FMA - SNOMED matching scenario. Specifically, the runtime of the second step has considerably increased and is comparable to the runtime of the first step. This can be explained by the worst-case time complexity of the McVitie and Wilson’s algorithm [39], that has been used, which is $\mathcal {O}\left (n^{2}\right)$. Moreover, the computation of the preference matrix required for defining the stable marriage assignment problem’s instance has worst-case time complexity *Θ*(*n*^2^ log*n*). At the same time, the space complexity of the second step is $\mathcal {O}\left (n^{2}\right)$, since it requires the storage of the preference matrices. On the contrary, various techniques [71, 72] and frameworks [69, 70, 73, 74] have been proposed and implemented for distributing the training and inference task of DNRs. Although our implementation exploits these highly optimized frameworks for DNRs, the choice of using the McVitie and Wilson’s algorithm introduces a significant performance barrier for aligning larger ontologies than the ones considered in our experiments. However, it was recently shown that a relationship exists between the class of computing greedy weighted matching problems and the stable marriage problems [75]. The authors exploit this strong relationship to design scalable parallel implementations for solving large instances of the stable marriage problems. It is among our future work to test the effectiveness of those implementations as well as to experiment with different graph matching algorithms that will offer better time and space complexity.

### Importance of the ontology extracted synonyms

As described in “Semantic lexicons” section, apart from the synonymy information extracted from ConceptNet 5, BabelNet, and WikiSynonyms, we have exploited the fact that, in some of the considered ontologies, a type may have one preferred name and some additional paraphrases expressed through multiple rdfs:label relations. In this section, we provide an additional set of experiments that aims to measure the importance of these extracted synonyms. This extracted synonymy information constitutes the 0.008*%*, 0.26*%*, 0.65*%* of the training data used in the MA - NCI, FMA - NCI, FMA - SNOMED matching scenarios, respectively. The high variance in their contribution to the training data provide us a means for partially evaluating the correlation between the relative change in the training data and the F1-score.

**Fig. 6:**
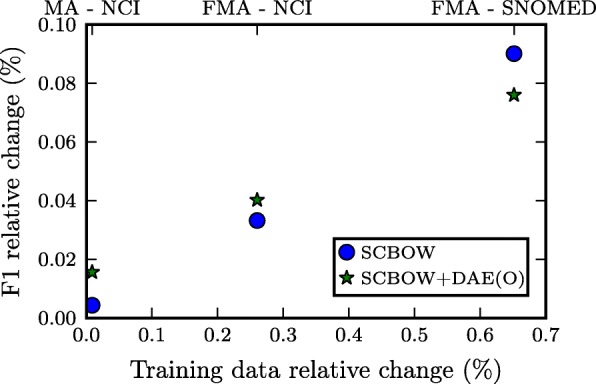
Correlation between the relative change in training data’s size and *F*1-score

In Table 6, we compare the performance of SCBOW and SCBOW+DAE(O) trained with only the available information from the semantic lexicons, with that presented in Table 2 where all the the synonymy information was available. It can be observed that the additional synonymy information affects positively both SCBOW and SCBOW+DAE(O). To better illustrate this correlation, we present in Fig. 6 how the relative change in the training data is reflected to the relative difference in the performance of our algorithm. It transpires that the F1-score’s relative change monotonically increases with the relative difference in the available data. This behavior constitutes a consistency check for our proposed method, since it aligns with our intuition that increasing the synonymy information leads to producing terminological embeddings more robust to semantic similarity. Regarding the additional benefit that this additional synonymy information brings, a maximum gain of 0.07 in the F1-score is observed across all the matching scenarios. This fact provides supplementary empirical support on the adequacy of the used general semantic lexicons as a means of providing the semantic similarity training data needed by our method. Although this additional synonymy information is important for comparing favorably with the state-of-the-art systems, it does not constitute a catalytic factor for the method’s success.

**Table 6:** Proposed algorithm’s performance in relation to the used synonymy information sources

System	Training data	MA - NCI			FMA - NCI			FMA - SNOMED		
		P	R	F1	P	R	F1	P	R	F1
SCBOW	SL	0.845	0.911	0.877	0.897	0.840	0.868	0.795	0.773	0.784
SCBOW	SL + AS	0.847	0.917	0.881	0.899	0.895	0.897	0.843	0.866	0.855
SCBOW + DAE(O)	SL	0.946	0.905	0.925	0.972	0.830	0.895	0.912	0.759	0.829
SCBOW + DAE(O)	SL + AS	0.968	0.913	0.94	0.976	0.892	0.932	0.931	0.856	0.892

Note: SL: synonyms only from ConceptNet 5, BabelNet, and WikiSynonyms; AS: additional synonyms found in the ontologies to be matched

Nonetheless, further experimentation is needed to verify the adequacy of these general semantic lexicons as well as to investigate the correlation between the training data size and the proposed method’s performance. We leave for future work the further experimentation with supplementary matching scenarios, different training data sizes and synonymy information sources.

### Threshold sensitivity analysis

**Fig. 7:**
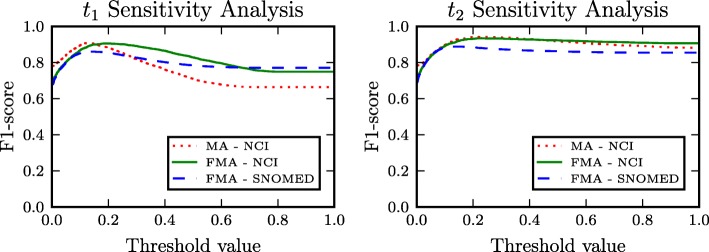
Sensitivity analysis of the proposed algorithm’s performance with different threshold values

In this section, we perform a sensitivity analysis for the thresholds *t*_1_ and *t*_2_. These thresholds constitute a means for quantifying if two terms are semantically similar or descriptively associated. It is worth noting that the tuning of these thresholds can be decoupled. Equivalently, the *t*_1_ threshold can be tuned to optimize the performance of SCBOW, and based on the resulted value the tuning of *t*_2_ can be performed so as to optimize the performance of the outlier detection mechanism. Figure 7 shows a threshold sensitivity analysis of our method. For exploring the effect of *t*_1_, we present on the left sub-figure of Fig. 7 the performance of SCBOW for all the different matching scenarios when varying the value of threshold *t*_1_ between 0 and 1.0. Similarly, the right sub-figure of Fig. 7 shows the performance of SCBOW+DAE(O) when *t*_1_ is set to 0.2 and the value of *t*_2_ varies in [ 0,1.0].

To begin with, it can be seen that both of the threshold sensitivity analysis’ figures undergo analogous qualitative behavior across the different ontology matching tasks. At the same time, it is observed that the performance (F1-score) monotonically increases when the value of *t*_1_ varies between 0 and approximately 0.2. In the *t*_1_ sub-figure, the performance monotonically decreases with *t*_1_∈ [ 0.2,0.6] and reaches an asymptotic value at about 0.6. In the case of *t*_2_, although the performance decreases when the value of *t*_2_ exceeds 0.2, the rate of the decrease is significantly lower compared to the rate of decrease of *t*_1_.

It can be seen that although further tuning and experimentation with the values of *t*_1_, *t*_2_ can give better results for each ontology matching task, the values that resulted from the hyperparameter tuning (described in “Training” section) are significantly close to the optimal ones. Moreover, it can be concluded that *t*_1_ values greater than 0.2 have a greater negative impact on the performance compared to the performance drop when *t*_2_ exceeds 0.2. Finally, it should be highlighted that apart from the hyperparameter tuning, no additional direct supervision based on the ground truth alignments is used by our method when we align the ontologies of the considered matching scenarios.

### Implications & limitations

Traditionally, ontology matching approaches have been based on feature engineering in order to obtain different measures of similarity [27]. This plethora of multiple and complementary similarity metrics has introduced various challenges including choosing the most appropriate set of similarity metrics for each task, tuning the various cut-off thresholds used on these metrics, etc. [76]. As a solution to these challenges, various sophisticated solutions have been proposed such as automating the configuration selection process by applying machine learning algorithms on a set of features extracted from the ontologies [76]. Unlike in our approach, only one similarity distance is used; the cosine distance upon the learned features of the phrase retrofitting and the DAE framework. Therefore, there is a drastic decrease in the used similarity metrics and thresholds.

At the same time, it was an open question whether ontology’s structural information is really required for performing ontology matching. Our proposed algorithm manages to compare favorably against state-of-the-art systems without using any kind of structural information. Our results support that a great ontology matching performance can be achieved even in the absence of any graph-theoretic information. However, we avoid to conclude that structural information is not necessary. We leave for future work the investigation of how the ontology’s structural information can be exploited in the frame of DNRs. Similarly, our method relies on word vectors pre-trained on large external corpora and on synonymy information provided by semantic lexicons also including the ontologies to be matched. Consequently, we can make the conclusion that external corpora and semantic lexicons provide sufficient information to perform ontology matching by only exploiting the ontologies’ terms.

Nonetheless, our approach has also certain shortcomings. To begin with, our proposed algorithm is restricted on discovering one-to-one correspondences between two ontologies. At the same time, the use of the McVitie and Wilson’s algorithm in our current implementation introduces a significant performance barrier for aligning lager ontologies than the ones considered in our experiments. Although our experimental results demonstrated that high precision can be achieved without using the OWL’s reasoning capabilities, our recall remains lower compared to the state-of-the-art systems across all the ontology matching tasks. Taking into account the results presented in “Importance of the ontology extracted synonyms” section, it may be concluded that more synonymy information is required to be extracted from supplementary semantic lexicons so as to increase this performance metric. This observation introduces another one weakness of our algorithm; that of closely depending on available external corpora and semantic lexicons. All the aforementioned open questions and shortcomings demonstrate various interesting and important directions for our future work and investigation.

## Related work

**Representation Learning for Ontology Matching:** Ontology matching is a rich research field where multiple and complementary approaches have been proposed [7, 77]. The vast majority of the proposed approaches, applied on the matching scenarios used in this paper, perform ontology matching by exploiting various terminological and structural features extracted from the ontologies to be matched. In parallel, they make use of various external semantic lexicons such as Uberon, DOID, Mesh, BioPortal ontologies and Wordnet as a means for incorporating background knowledge useful for discovering semantically similar terms. CroMatcher [64], AML [60, 61] and XMap [65] extract various sophisticated features and use a variety of the aforementioned external domain-specific semantic vocabularies to perform ontology matching. Moreover, LogMap, AML and XMap exploit complete and incomplete reasoning techniques so as to repair incoherent mappings [78]. Unlike the aforementioned approaches, FCA_Map [66, 67] uses Formal Concept Analysis [68] to derive terminological hierarchical structures that are represented as lattices. The matching is performed by aligning the constructed lattices taking into account the lexical and structural information that they incorporate. PhenomeNet [79] exploits an axiom-based approach for aligning ontologies that make use of the PATO ontology and Entity-Quality definition patterns [80, 81]; complementing in that way some of the shortcomings of feature-based methods.

Representation learning has so far limited impact on ontology matching. To the best of our knowledge, only two approaches, [82–84], have explored so far the use of unsupervised deep learning techniques. Both of these approaches use a combination of the class ID, labels, comments, etc. to describe an ontological entity in their algorithms. Zhang et al. [82] are the first ones that investigated the use of word vectors for the problem of ontology matching. They align ontologies based on *word2vec* [14] vectors trained on Wikipedia. They were the first that reported that the general-purpose word vectors were not good candidates for the task of ontology matching. Xiang et al. [83, 84] proposed an entity representation learning algorithm based on Stacked Auto-Encoders [37, 85]. However, training such powerful models with so small training sets is problematic. We overcome both of the aforementioned problems by using a transfer learning approach, known to reduce learning sample complexity [86], which retrofits pre-trained word vectors to a given ontological domain.

**Sentence Representations from Labeled Data:** To constrain the analysis, we compare neural language models that derive sentence representations of short texts optimized for semantic similarity based on pre-trained word vectors. Nevertheless, we consider in our comparison the initial Siamese CBOW model [32]. Likewise, we do not focus on innovative supervised sentence models based on neural networks architectures with more than three layers including [87, 88] and many others. The most similar approach to our extension on Siamese CBOW is the work of Wieting et al. [22]. Wieting et al. address the problem of paraphrase detection where explicit semantic knowledge is also leveraged. Unlike in our approach, a margin-based loss function is used, and negative examples should be sampled at every step introducing an additional computational cost. The most crucial difference is that this model was not explicitly constructed for alleviating the coalescence of semantically similar and descriptively associated terms. Finally, the initial Siamese CBOW model was conceived for learning distributed representations of sentences from unlabeled data. To take advantage of the semantic similarity information already captured in the initial word embeddings, an important characteristic as demonstrated in various word vectors retrofitting techniques [20–22], we extended the initial model with an knowledge distillation reguralizer. Finally, we further extended the initial softmax setting, with a tempered softmax, with the purpose of enabling the network to capture information hidden in small logit values.

**Autoencoders for Outlier Detection:** Neural networks applications to the problem of outlier detection have been studied for a long time [89, 90]. Autoencoders seem to be a recent and a very prominent approach to the problem. As has been pointed out in [91], they can be seen as a generalization of the class of linear schemes [92]. Usually, the reconstruction error is used as the outlier score [91]. Recently, Denoising Autoencoders (DAEs) have been used for outlier detection in various applications, such as acoustic novelty detection [93], network’s intrusion detection [91], anomalous activities’ discovery in video [94]. To the best of our knowledge, this is the first time that the problem of semantic similarity is seen from the viewpoint of outlier detection based on DAEs. Unlike the other approaches, we want to detect outliers in pairs of input. To achieve that we use the cosine distance over the two produced hidden representations as an outlier score, instead of using the reconstruction error which is customary in the literature. Our motivation is that intrinsic characteristics of the distribution of semantically similar terms are captured in the hidden representation and their cosine distance could serve as an adequate outlier score. Unlike the majority of the aforementioned work, we do not train end-to-end the DAE but we follow a layer-wise training scheme based on sentence representations produced by our extension of Siamese CBOW. Our impetus is to let the DAE to act on a dataset with significant less noise and bias.

## Conclusions

In this paper, we address the problem of ontology matching from a representation learning perspective. We propose the refinement of pre-trained word vectors so that when they are used to represent ontological terms, the produced terminological embeddings will be tailored to the ontology matching task. The retrofitted word vectors are learned so that they incorporate domain knowledge encoded in ontologies and semantic lexicons. We cast the problem of ontology matching as an instance of the Stable Marriage problem using the terminological vectors’ distances to compute the preference matrix. We compute the aforementioned distances using the cosine distance over the terminological vectors learned by our proposed phrase retrofitting process. Finally, an outlier detection component, based on a denoising autoencoder, sifts through the list of the produced alignments so as to reduce the number of misalignments. Our experimental results demonstrate significant performance gains over the state-of-the-art and indicate a new pathway for ontology matching; a problem which has been traditionally studied under the setting of feature engineering.

## Footnotes

1 This term is known in the NLP community as “conceptually associated”. We have chosen to depart from the standard terminology for reasons summarized in [95, p. 7].

2 We provide further justification for this choice in “Evaluation benchmarks” section.

3 We provide further details on the textual information used in our experiments in “Evaluation benchmarks” section.

4 available on OAEI’s 2016 Large BioMed Track.

5
http://oaei.ontologymatching.org/2016/

6 For a detailed overview and comparison of the systems refer to [96].

7 We have also performed hyperparameter tuning in the SNOMED-NCI matching task, which gave the same hypeparameters as the ones reported in [23].

8 except for the synonymy information found in some ontologies and is expressed through multiple labels (rdfs:label) for a given type.

9 All the experiments are statistically significant with a *p*-value ≤ 0.05.
